# Coronary Artery Aneurysm Post Drug-Coated Balloon Angioplasty

**DOI:** 10.1016/j.jaccas.2025.103891

**Published:** 2025-07-03

**Authors:** Aninka Saboe, Chetan Upadhya, Aloke Finn, Sandeep Basavarajaiah

**Affiliations:** a Universitas Padjadjaran–Hasan Sadikin General Hospital, Indonesia; b Glan Clwyd Hospital, Bodelwyddan, Denbigshire, North Wales, United Kingdom; c CVPath Institute, Gaithersburg, Maryland, USA; d Heartlands Hospital, University Hospital Birmingham, United Kingdom

**Keywords:** acute coronary syndrome, drug-coated balloon, percutaneous coronary intervention

## Abstract

Drug-coated balloons have emerged as effective therapy for treating in-stent restenosis and de novo lesions. The animal studies have shown the mechanism of action of paclitaxel-coated balloon in coronary arteries which leads to medial necrosis that may explain the better angiographic remodelling observed with paclitaxel- as compared to sirolimus-coated balloons. However, in some cases, paclitaxel may cause adverse remodelling resulting in coronary artery aneurysm. Here, we report 4 cases of coronary artery aneurysm post paclitaxel-coated balloon angioplasty and discuss its possible mechanism, management, and potential implications for future practice.

## Graphical Abstract

**Figure:**
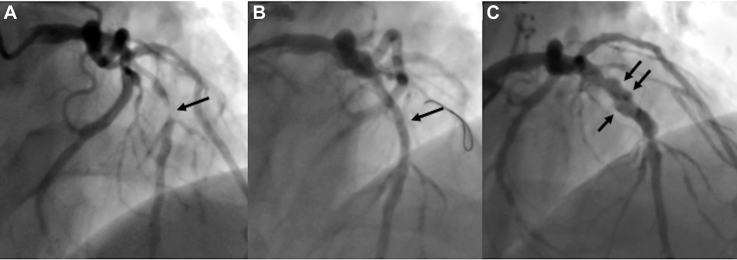

Drug-coated balloons (DCBs) are becoming increasingly popular in percutaneous coronary interventions for use in both in-stent restenosis and de novo lesions due to consistent trickle of positive data.^1^, ^2^, ^3^ They provide the benefit of local drug delivery without leaving a permanent implant; hence, they offer advantages over drug-eluting stents (DES) in maintaining original artery anatomy eliminating the risk of stent thrombosis and shorter dual antiplatelet therapy (DAPT). However, despite their benefits, certain complications have been reported including the development of coronary artery aneurysms (CAAs). A CAA is defined as a localized dilation of a coronary artery exceeding 1.5 times the diameter of adjacent normal segments. Although traditionally linked with atherosclerosis and Kawasaki disease, the use of DCB, especially the paclitaxel-coated balloon, has raised concerns about iatrogenic CAA. The management of these conditions poses a clinical dilemma to the clinicians due to lack of data from trials or recommendations. We report 4 clinical cases of CAA post DCB angioplasty and discuss possible mechanisms and management strategies in dealing with such a complication.

**Take-Home Messages**

• Aneurysm post therapy with drug-coated balloons is rare but more often reported following the use of paclitaxel-coated balloons which might be related to a possible mechanism of action. Paclitaxel has cytotoxic properties that may make the vessel prone to exaggerated or abnormal remodelling leading to ectasia or aneurysm.
• However, despite this, paclitaxel has shown safety and efficacy when used in a balloon unlike in a stent platform.
• The emergence of sirolimus-coated balloons for therapy has given the alternative options for the operators especially when the Limus drug is gentler on arteries with cytostatic action unlike paclitaxel.

## Case 1

**Figure 1:**
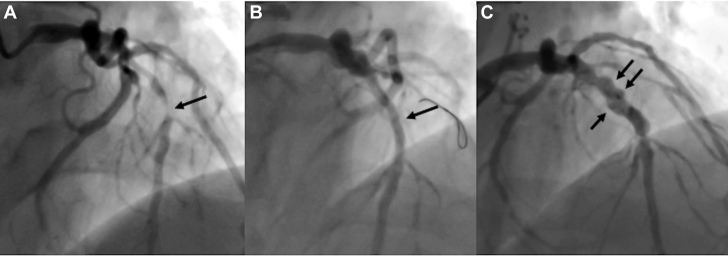
Coronary Angiogram of Patient 1 (Case 1) Pre-PCI, Post-PCI and During Follow-Up (A) Occlusion of the left anterior descending artery from the mid-segment (arrow). (B) Angiogram after successful treatment with a paclitaxel-coated balloon (arrow). (C) At 6-month follow-up, a coronary artery aneurysm is shown (arrows).

A 57-year-old man with acute coronary syndrome (ACS) and a background of hypertension was found to have a severe stenosis in the mid segment of the left anterior descending (LAD) artery (Figure 1A). Percutaneous coronary intervention was performed by predilating the lesion with a 3.0 × 15-mm semi-compliant balloon and a 3.5 × 15-mm scoring balloon. Angiogram post-predilatation demonstrated excellent results with no significant recoil or dissection. Given his young age, the lesion was treated with a 3.5 × 30-mm paclitaxel-coated balloon (PCB) to achieve good final result and he was discharged 72 hours post-admission without any complications (Figure 1B, Video 1). The patient underwent a follow-up angiogram 6 months post-procedure which revealed an aneurysmal dilation in the previously treated segment (Figure 1C, Video 2) with TIMI flow grade 3. Because the patient was asymptomatic, conservative management was planned with continuation of DAPT and a surveillance computed tomography angiography (CTA) in 6 months.

## Case 2

**Figure 2:**
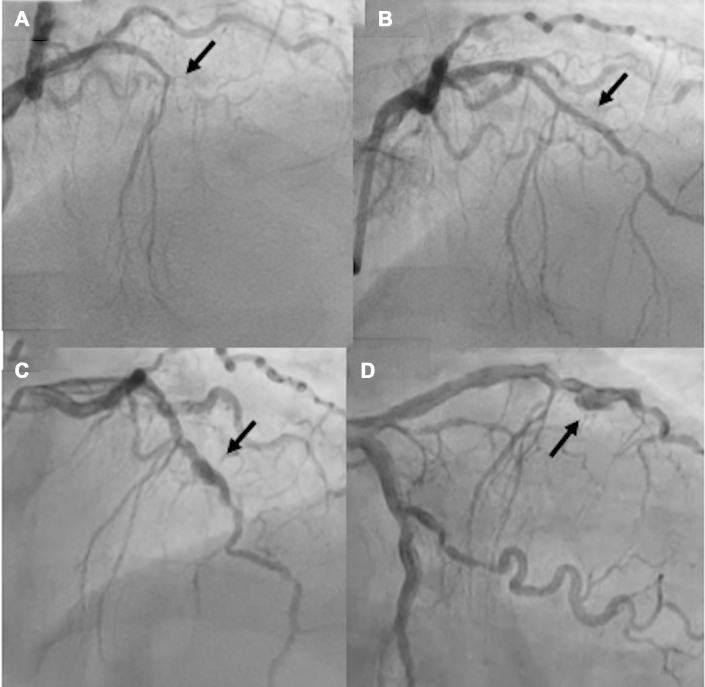
Coronary Angiogram of Patient 2 (Case 2) Pre-PCI, Post-PCI and During Follow-Up (A) Occlusion of the left anterior descending artery from the mid-segment (arrow). (B) After successful treatment with a paclitaxel-coated balloon (arrow). (C, D) Coronary angiogram in the cranial and caudal views at 6 months follow-up (arrow).

A 65-year-old female with a background of hypertension presented with acute antero-lateral myocardial infarction. The angiogram showed total occlusion in the mid-segment of the LAD with TIMI flow grade 0 (Figure 2A). The occlusion was treated with a 2.5 × 15-mm semicompliant balloon which established TIMI flow grade 3 with minimal residual stenosis and no significant dissection. Because the vessel was small, it was treated with a 2.5 × 25-mm PCB to achieve excellent final angiographic result (Figure 2B, Video 3). The follow-up angiogram at 6-months post-procedure revealed aneurysmal dilation in the distal LAD (Figures 2C and 2D, Video 4) with TIMI flow grade 3. Because the patient was asymptomatic, conservative management was planned, and DAPT was continued with scheduled follow-up at 6 months with CTA.

## Case 3

**Figure 3:**
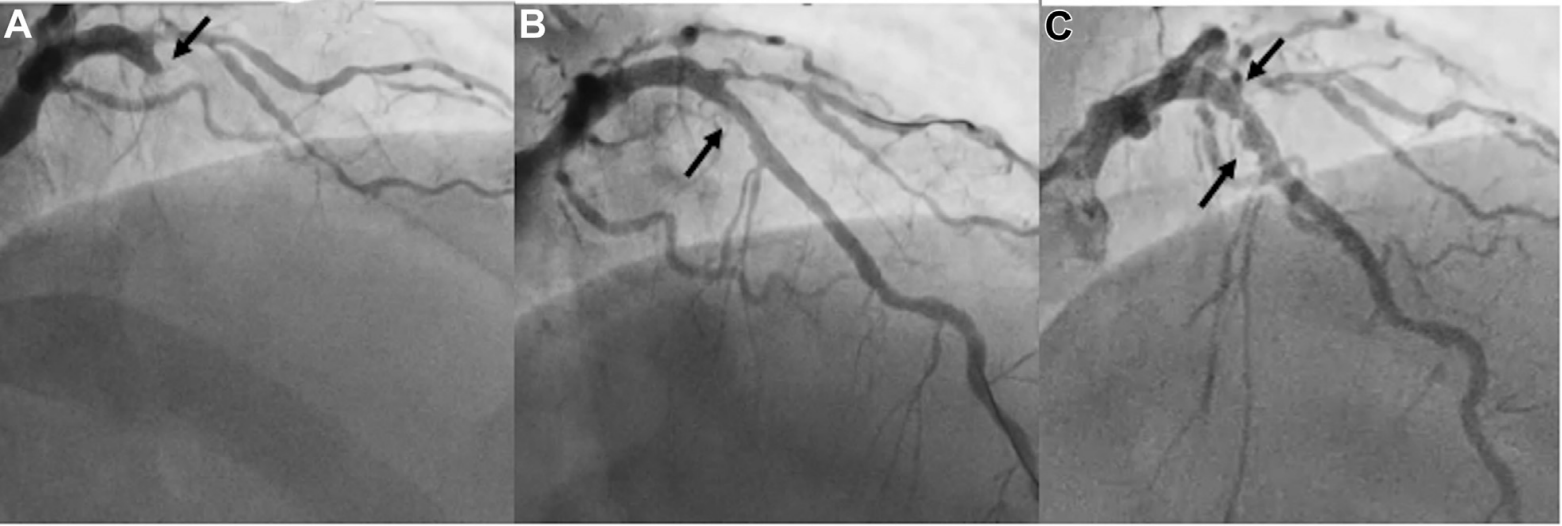
Coronary Angiogram of Patient 3 (Case 3) Pre-PCI, Post-PCI and During Follow-Up (A) Occlusion of the left anterior descending artery from the proximal segment (arrow). (B) After successful treatment with a paclitaxel-coated balloon (arrow). (C) Six-month follow-up showing coronary artery aneurysm (arrows).

A 44-year-old male with anterior wall myocardial infarction was found to have an occluded LAD from mid-segment with TIMI dlow grade 0 (Figure 3A). Following wiring of the LAD, predilation was performed with a 3.5 × 15-mm semicompliant balloon, which established TIMI flow grade 3. Given his young age, we wanted to avoid use of a DES; therefore, we used a 3.5 × 15-mm PCB (Figure 3B, Video 5). The 6-month follow-up angiogram showed an aneurysmal dilation in the same segment of the LAD that was treated with a PCB (Figure 3C, Video 6). Because the patient was asymptomatic, the aneurysm was managed conservatively by continuing the DAPT with a planned angiogram follow-up.

## Case 4

**Figure 4:**
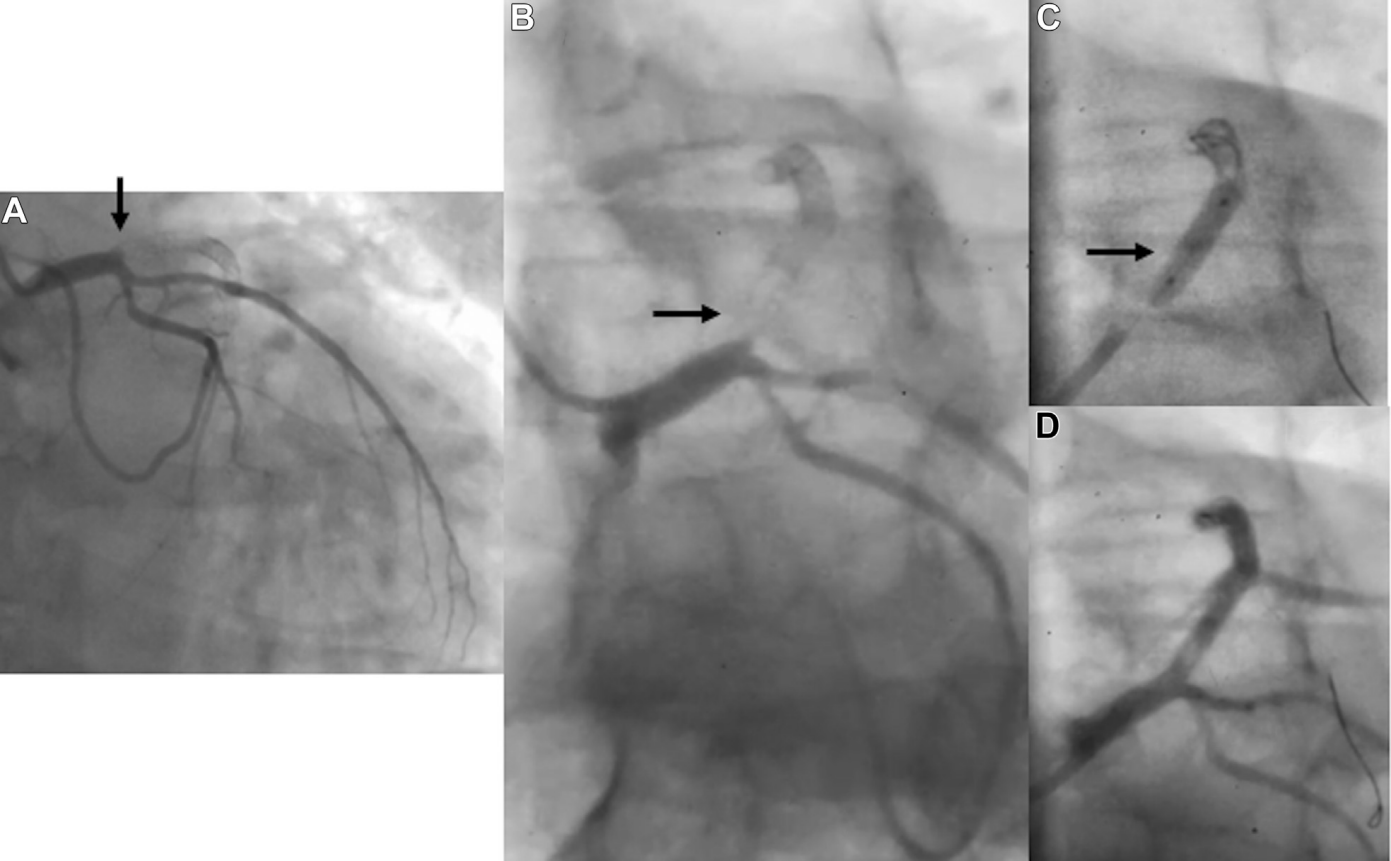
Coronary Angiogram of Patient 4 (Case 4) Pre-PCI, Post-PCI and During Follow-Up (A, B) Occlusion shown from the proximal segment within the previously placed stent (arrow). (C, D) After successful treatment with a paclitaxel-coated balloon (arrows).

**Figure 5:**
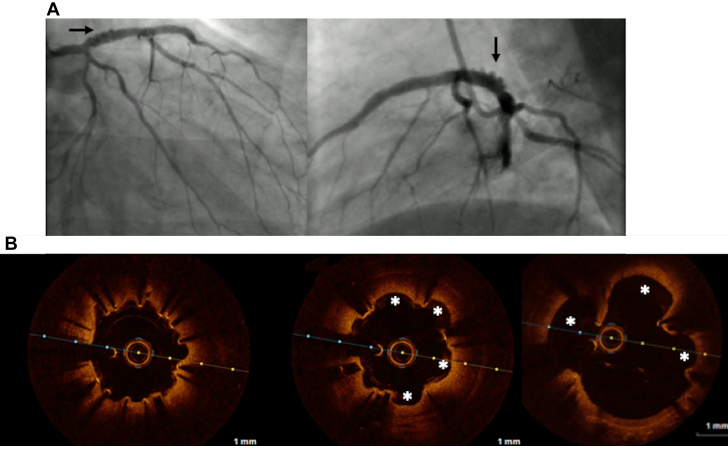
OCT of Patient 4 (Case 4) Performed During Follow-Up Angiogram (A) Coronary angiogram and (B) optical coherence tomography (OCT) showing coronary artery aneurysm (shown by arrows and stars, respectively).

A 65-year-old man who had received 2 DES in the proximal and mid-segment of the LAD, respectively, in 2010 re-presented in 2022 with acute anterior wall myocardial infarction. The LAD was found to be occluded in its proximal segment (Figures 4A and 4B). After establishing TIMI flow grade 3 with thrombus aspiration and balloon dilatation, intravascular imaging was performed which revealed a thrombotic lesion in the proximal LAD, but the previous placed stents were well apposed. To avoid another layer of metal, a PCB was used mainly in the proximal segment (Figures 4C and 4D, Video 7). The patient had a follow-up angiogram for recurrence of chest pain at 6 months which showed a new finding of aneurysms in the proximal LAD where the DCB was used, and this was confirmed by optical coherence tomography (Figures 5A and 5B, Videos 8 and 9). While the case was being discussed to offer appropriate treatment, the patient succumbed to sudden cardiac death. We attributed his sudden death to either an acute occlusion from thrombus formation within the aneurysm or fatal arrhythmia from his underlying left ventricular dysfunction.

## Discussion

CAA is characterized by abnormal coronary artery dilation exceeding the diameter of adjacent healthy segments by at least 50%. Although the exact mechanism behind the development of spontaneous CAA is still unclear, several theories have been proposed including those suggesting infection, smoking, or atherosclerosis.^4^ However, CAA occurring post angioplasty is attributed to the procedure and the device used. The potential contributing factors include significant acute vessel injury during the initial procedure, hypersensitivity reactions, and abnormal vessel remodelling triggered by drug and/or polymer.^5^^,^^6^ In the era of first-generation DESs, the paclitaxel in combination with polymer that was used to elute the drug caused inflammation resulting in abnormal remodelling.^6^ However, this phenomenon has not been observed with the current-generation DES because the paclitaxel on stents are obsolete and the polymer used are biocompatible. Paclitaxel remains the drug of choice on balloons because of its high lipophilicity that results in better drug transfer and better angiographic remodelling than sirolimus. Paclitaxel has antiproliferative biological effects that exert smooth muscle cells, endothelial cells, and fibroblasts after transfer into the vessel wall. Use of paclitaxel leads to reduction in neointimal growth and is accompanied by persistent fibrin deposition, macrophage infiltration, and an overall decrease in smooth muscle cells impairing vessel healing and promoting inflammation.^7^ Unlike sirolimus, paclitaxel is a cytotoxic drug. Recent animal model studies have shown medial necrosis in the segments treated with PCB which may explain the better vessel remodelling observed post PCB than after use of sirolimus-coated balloons.^8^ The reports of aneurysm post DCB have almost been exclusively observed in PCB cases; this might be due to exaggerated or adverse remodelling from the mechanism of action described in the earlier section. Despite these effects, PCBs have demonstrated safety and efficacy since their inception in 2005 and paclitaxel remains the popular drug of choice on balloons.^9^ However, witnessing these findings poses a dilemma as to whether such aneurysm formation has any clinical significance. Certainly, the last patient presented (case 4) suffered sudden death, which could have been from fatal arrhythmias from his left ventricular dysfunction or equally from stent thrombosis that might have originated in the pockets of aneurysm. The first 3 patients have remained asymptomatic but are being followed up with CTA to monitor the progress of the aneurysms. All 4 cases treated with PCBs were in the setting of ACS, which implies that there may be a correlation with development of abnormal remodelling and ACS. The active inflammation observed at the lesion site during ACS may be augmented by paclitaxel given his cytotoxic properties; hence, they may make the vessel vulnerable for such abnormal remodelling. The risks posed by such an aneurysm are either vessel thrombosis or even rupture. It is also not clear whether such CAA post PCB is progressive, static, or even regressive, especially once the trigger (paclitaxel) disappears. One of the potential treatment options are to place covered stents to isolate the aneurysms from the mainstream circulation, thereby reducing the risk of thrombosis and rupture. However, the covered stents by themselves are prone to restenosis and stent thrombosis. Although PCB offers a valuable alternative to stent implantation in specific coronary pathologies, the development of CAA represents a potentially significant complication. Long-term angiographic follow-up data post PCB are lacking regarding efforts to understand the actual incidence of CAA post DCB. Further research is needed to understand the mechanisms underlying CAA formation after DCB use and to determine optimal treatment strategies. It is unclear which patients and/or lesion subsets are prone to such abnormal remodelling at this stage. Should we be shifting to Limus-coated balloons (which have a wider margin of safety)? Are we witnessing the emergence of data on the efficacy from sirolimus-coated balloons in clinical trials?^10^^,^^11^ Further research is necessary to determine these important answers.

## Funding Support and Author Disclosures

The authors have reported that they have no relationships relevant to the contents of this paper to disclose.

## Appendix

Video 1 Coronary angiogram after successful treatment with a paclitaxel-coated balloon (arrow).

Video 2 Coronary angiogram at 6-month follow-up showing coronary artery aneurysm (arrows).

Video 3 Coronary angiogram after successful treatment with a paclitaxel-coated balloon (arrow).

Video 4 Coronary angiogram in the anteroposterior (AP) cranial and AP caudal views at 6-month follow-up showing coronary artery aneurysm (arrow).

Video 5 Coronary angiogram after successful treatment with a paclitaxel-coated balloon (arrow).

Video 6 Coronary angiogram at 6-month follow-up showing coronary artery aneurysm (arrows).

Video 7 Coronary angiogram after successful treatment with a paclitaxel-coated balloon (arrows).

Video 8 Coronary angiogram and optical coherence tomography showing coronary artery aneurysm (arrows and stars, respectively).

Video 9 Coronary angiogram and optical coherence tomography showing coronary artery aneurysm (arrows and stars, respectively).
